# A-WEAR Bracelet for Detection of Hand Tremor and Bradykinesia in Parkinson’s Patients

**DOI:** 10.3390/s21030981

**Published:** 2021-02-02

**Authors:** Asma Channa, Rares-Cristian Ifrim, Decebal Popescu, Nirvana Popescu

**Affiliations:** 1 Computer Science Department, University POLITEHNICA of Bucharest, RO-060042 Bucharest, Romania; asma.channa@stud.acs.upb.ro (A.C.); rares.ifrim@stud.acs.upb.ro (R.-C.I.); decebal.popescu@upb.ro (D.P.); 2 DIIES Department, University Mediterranea of Reggio Calabria, 89100 Reggio Calabria, Italy

**Keywords:** Parkinson’s disease, tremor, bradykinesia, wearable technology, bracelet, machine learning

## Abstract

Parkinson’s disease patients face numerous motor symptoms that eventually make their life different from those of normal healthy controls. Out of these motor symptoms, tremor and bradykinesia, are relatively prevalent in all stages of this disease. The assessment of these symptoms is usually performed by traditional methods where the accuracy of results is still an open question. This research proposed a solution for an objective assessment of tremor and bradykinesia in subjects with PD (10 older adults aged greater than 60 years with tremor and 10 older adults aged greater than 60 years with bradykinesia) and 20 healthy older adults aged greater than 60 years. Physical movements were recorded by means of an AWEAR bracelet developed using inertial sensors, i.e., 3D accelerometer and gyroscope. Participants performed upper extremities motor activities as adopted by neurologists during the clinical assessment based on Unified Parkinson’s Disease Rating Scale (UPDRS). For discriminating the patients from healthy controls, temporal and spectral features were extracted, out of which non-linear temporal and spectral features show greater difference. Both supervised and unsupervised machine learning classifiers provide good results. Out of 40 individuals, neural net clustering discriminated 34 individuals in correct classes, while the KNN approach discriminated 91.7% accurately. In a clinical environment, the doctor can use the device to comprehend the tremor and bradykinesia of patients quickly and with higher accuracy.

## 1. Introduction

Despite the large available literature regarding the diagnosis and evaluation of tremor and bradykinesia and recognition of their severity level, there is still a need for a throrough investigation. In order to fill this gap, this research study expounds upon introducing a wearable wrist worn device in parallel with a machine learning (ML) algorithm that can objectively detect tremor and bradykinesia and also automatically measures the exact features related to the Unified Parkinson’s Disease Rating Scale (UPDRS). The main objective of this paper is to change the scenario of clinical diagnosis of tremor and bradykinesia by developing a wearable bracelet that collects data from the subjects on which an ML approach will be used in order to attain a more accurate and sophisticated diagnosis, which will eventually be of clinical convenience to patients and doctors.

### 1.1. Background

Parkinson’s disease (PD) is a neurodegenerative disease which is increasing globally and has significant impact on quality of life (QoL) [1]. The cardinal motor symptoms in PD patients are tremors, bradykinesia, freezing of gait (FOG), rigidity and postural instability [2]. A tremor is defined as an involuntary and rhythmic shaking. Bradykinesia is a slowness of movement and is mostly related to muscle weakness, rigid muscles, or tremors. According to [3], the tremor is one of the most visible and bothersome symptoms of PD patients. Tremor in PD patients creates negative affects and destroys patients’ self-image, sense of security, and well-being, as the patient is not able to write correctly with help of typewriter, fix small things, eat or hold the book for reading. According to the survey article [4], tremor is among the top three PD-related challenges that patients have reported to date. Around 70% of the PD patients notice tremors as the early symptom [5].

Although the recurrence of tremor is highly linked to PD, the extent of bradykinesia is much higher in advanced phases of the disease [6] compared to early stages. With respect to the performance of daily life activities and particularly, with upper limb function, the patient faces enormous difficulty because of tremor and bradykinesia, which certainly decrease the QoL of the PD patient. It is one of the challenges for researchers and clinicians to implement a system that helps them to evaluate and provide an unbiased tracking of these cardinal motor symptoms of the PD population in a free living environment where they can easily perform the meaningful tasks. The tremor is an involuntary quivering movement or shake and is characterized as the most common movement disorder. The one observed in PD patients is known as the rest tremor (RT) or the Parkinsonian tremor. It unpretentiously occurs at rest, classically slow and usually begins in one hand or leg, but sooner or later affects both sides of the body. The different types of tremor, i.e., essential tremor, Parkinsonian tremor and dystonia tremor are very common among PD patients [7]. Table 1 explains the key features of these tremors that help clinicians to diagnose.

The clinical diagnosis of parkinsonian tremor and bradykinesia is carried out using rating scales. There are a number of rating scales, such as UPDRS, Hoehn and Yahr Scale, Schwab and England Activities of Daily Living (ADL) Scale, PDQ-39, PD NMS Questionnaire and other scales. A rating scale is a way of providing information on a specific feature by assigning a value to it. Parkinson’s rating scale depends on the ‘rater’—the person who decides the points patients tally. Among all these clinical rating scales, UPDRS is the most adopted one, preferred by neurologists [8]. In context to [9,10], bradykinesia diagnosis requires experienced doctors, but sometimes they also miss subtle changes, especially in the early stages of the disease. Although the UPDRS method is used all over the world and is considered as the gold standard of PD diagnosis, its results are based on observations, which can be sometimes inaccurate. There is a dire need of a system that provides more validated results. These types of rating scales stand in need of a wearable device that is easy to wear and to remove, does not discommode in performing of tasks, requires less or no maintenance at all and calibration is done once in the field and is sensitive to a variety of relevant signs and symptoms.

### 1.2. Related Work

As already pointed out, the tremor is one of the relevant motor symptoms of PD that must be counted for the correct evaluation and diagnosis of disease and it has been widely addressed by [11,12,13,14,15,16,17,18,19,20,21,22,23,24,25,26,27,28,29,30,31]. Therefore, numerous studies have employed wearable sensors as a robust method for accurate findings. In [11,12,13,16,27,29,31], EMG signals are analyzed for tremor assessment and quantification. However, in [14,18,20,25], the smartphone is considered as a useful tool for the assessment of tremor. Table 2 offers a clear overview of the studies that used accelerometer and gyroscopes on body parts, particularly on the wrist and forerams, for diagnosis and interpretation of tremor. For instance, [32] used a smartwatch equipped with gyroscopes and quantified PD patients’ tremors using 64 smartwatch recordings. The monitoring for tremor intensity was 0.81 (*p* < 0.001); by using intraclass reliability coefficient reliability to quantify tremors with a resting tremor = 0.89. In [33], researchers used inertial devices and extracted 78 and 96 upper and lower limbs kinematic parameters to analyze motor performance in PD patients.

The presence of cardinal motor symptoms in PD patients is very common and research shows that the prevalence of this disease and motor disability increases with time in older PD adults, which gradually draws them to gait disturbances. Many researchers conducted the assessment of bradykinesia from the lower extremities of PD patients, as in [34,35,36,37]. However, in [15,38,39,40], various techniques were used in the detection and quantification of bradykinesia from upper-extremity body movements, as shown in Table 3. PD patients’ tremor and bradykinesia lie at extreme lower frequencies; hence, it is important to evaluate the minute changes in motor activities. Therefore, it is reported that inertial sensors works well for this.

In spite of all these studies, the adoption of different methodologies makes it even more difficult to determine which inertial sensor will be the best to use, at which position of body it should be attached and what features give optimal results in identification and evaluation. The main objective of this research study is to introduce an AWEAR bracelet developed using two inertial sensors, which are a 3D gyroscope and a 3D accelerometer to be used by neurologists in clinical assessment and for correct diagnosis of tremor and bradykinesia. The bracelet is attached at the wrist joint in order to obtain the perfect kinetic feature since, according to [41], PD initially affects the upper limbs of patients and the wrist helps coordinate most of the hand and arm movements. The study also addressed the temporal and spectral features which served as key features in distinguishing tremor and bradykinesia in PD patients from same-age healthy elderly adults.

## 2. Experimental Set-Up

### 2.1. Measurement System/Hardware Description

**Figure 1:**
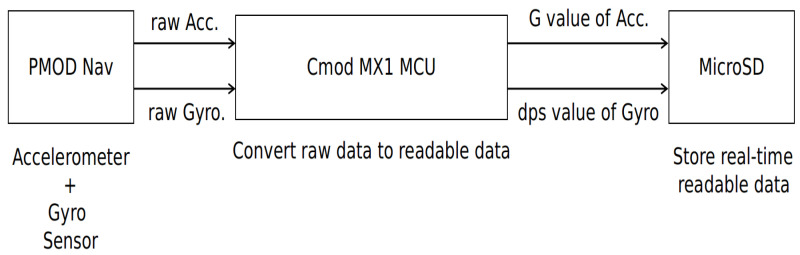
Block design for the bracelet.

In this study, we used a hand bracelet as a measurement unit. The bracelet acquires data about the hand movements of the patients and healthy participants. The bracelet is developed using a small form-factor microcontroller, that reads real-time data from a special sensor module that contains an accelerometer and a gyroscope and writes its output values to a micro SD card. Figure 1. shows the block design of the AWEAR bracelet.

The components used for this bracelet are:

1. The Cmod MX1 microcontroller containing a microchip PIC32MX150F128D microprocessor.
2. Pmod NAV module: 3-axis accelerometer and 3-axis gyroscope sensor.
3. Pmod micro SD module, which is used for storing data on the micro SD card.

**Figure 2:**
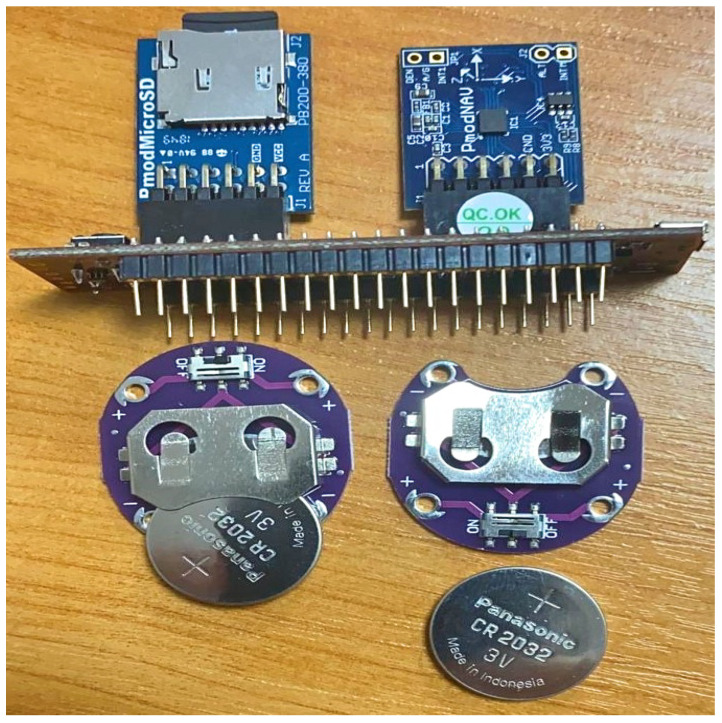
Components used for development of the AWEAR bracelet.

**Figure 3:**
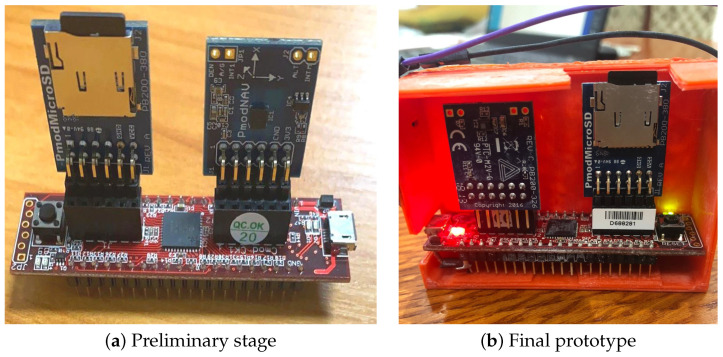
AWEAR bracelet developmental stages.

Figure 2 shows the components used for development of the bracelet. Here, Pmod NAV provides a variety of orientation related data, allowing users to easily determine the exact position the module is in and where it is headed. It gives raw data, and with the speed provided by the 40 MHz PIC microprocessor we can calculate the accelaration (G) and the degrees per second (dps) values of the accelerometer and gyroscope before storing them on the SD card, so that the neural and machine learning (ML) classifiers can work directly with the readable data, and do not waste time on converting the raw data. Both the Pmod NAV and the micro SD use the SPI (serial peripheral interface) protocol to talk with the microcontroller. The microcontroller acquires real time data for a fixed period of time, long enough so that we can have an accurate interpretation of the data. For the first version, we considered 2 status LEDs, one being for notifying the user that the bracelet was calibrated and all modules were correctly initiated. After the first LED turns ON, it means that the data acquisition has started. When the second LED turns on, it means that the data acquisition finished, and the micro SD card can be safely removed for data reading and analyzing. The bracelet is designed in a form so that it is easy for the patients to wear on wrist. We used two 3 V batteries mostly used in digital watches which lasts for 3 h. This bracelet is used only for collecting data. Figure 3a shows the preliminary developmental stage of bracelet and Figure 3b shows final prototype.

### 2.2. The Subjects and the Acquisition Procedure

A total of 40 subjects participated in this study, from which 20 subjects have PD with varying degrees of tremor and bradykinesia severity and the rest involved age matched healthy controls. Regarding the PD patients, 5 men and 15 women have been asked to participate in this study. Other details of them are: mean age ± standard deviation (SD): 71.65 ± 6.872 years old; average MDS/UPDRS scores ± SD: 18.91 ± 7.831; average Hoehn and Yahr (H &Y) stage ± SD: 1.65 ± 0.526 with disease duration in years ± SD: 7.7 ± 4.495. The set of healthy participants consists of 16 men and 4 women, having the mean ± SD: 70.25 ± 6.307 years old. Among 20 patients, 10 patients just have tremor symptom with no bradykinesia sign and 10 patients have bradykinesia symptom with no sign of tremor while performing the activities as in [42]. The most relevant demographic and clinical information of subjects is explained in Table 4. The acquisition procedure includes two sessions. In the first session, only those activities that helped in tremor detection were performed and in the second one, bradykinesia detection activities were realised. The data acquisition has been done in home environment due to COVID-19 measures, in the presence of a neurologist. All subjects recruited in this study signed an informed consent form and they were asked to perform the tasks accurately. Bracelet is affixed to the predominantly affected hand of patients.

#### 2.2.1. Part 1: Validation of Tremor Detection

The resting tremor is one of the most prominent tremors that occurs in PD patients, but 25% PD patients also face action and postural tremor. The severity increases when all of these types are present in PD patient.

1. Testing for Resting tremor (RT): To evaluate RT within the upper limits, the patients are inquired to rest the forearms comfortably on the thighs for one minute. Resting tremor most commonly shows up as a flexion-extension development of the wrist/hand, a pronation-supination exercise of the forearm, or a pill-rolling exercise of the thumb and index finger.
2. Testing for Postural tremor (PT): Postural tremor is a kind of tremor that develops when the patient maintains a position against gravity and its frequency is typically in between 4–12 Hz [43]. To test for postural tremor, the patient is first asked to completely elongate the elbow and to flex the arm forward at 90°. At that point, the subject is requested to spread their fingers out as much as conceivable and continue this position for a minute. This is essential since a PT in PD is often evidenced in a minute after the position is accepted.
3. Action or kinetic tremor (KT): This sort of tremor shows up only when the participant is carrying out an activity. The recurrence of kinematic tremor is often between 2–7 Hz [44]. To test for action tremor, the finger to nose test is considered. In performing this movement, the patients are taught to alternatively touch their nose and observer finger. In doing so, the patients ought to extend their arm fully and ought not to move quickly. In this way, we have more chance of activating the tremor. This test is performed for 60 s on each partcipant.

#### 2.2.2. Part 2: Validation of Bradykinesia Detection

Bradykinesia is also one of the cardinal motor symptoms of PD patients. In order to track it, some exercises should be followed by the patients.

1. Finger Tapping: The primary test is finger tapping in which the control subject is seated and requested to tap his thumb and index finger as much as he can and as quickly as feasible for 60 s.
2. Fist Open and Close: Bradykinesia is likewise rated with the arms in the same position as for hand movement, but this time inquiring the patient to open and close the hand as fast as feasible, along with the biggest possible excursion. This activity is attempted for one minute.
3. Pronation/Supination: Bradykinesia is also rated for each upper extremity by asking the seated patient to raise the elbow to the level of the mid-chest, flex it to 90° with the hand pointing up, and after that move the hand and forearm as fast as feasible with the greatest possible excursion. This motion is continued for 60 s. This is often related in the same way as finger tapping for each side.

## 3. Methodology

**Figure 4:**
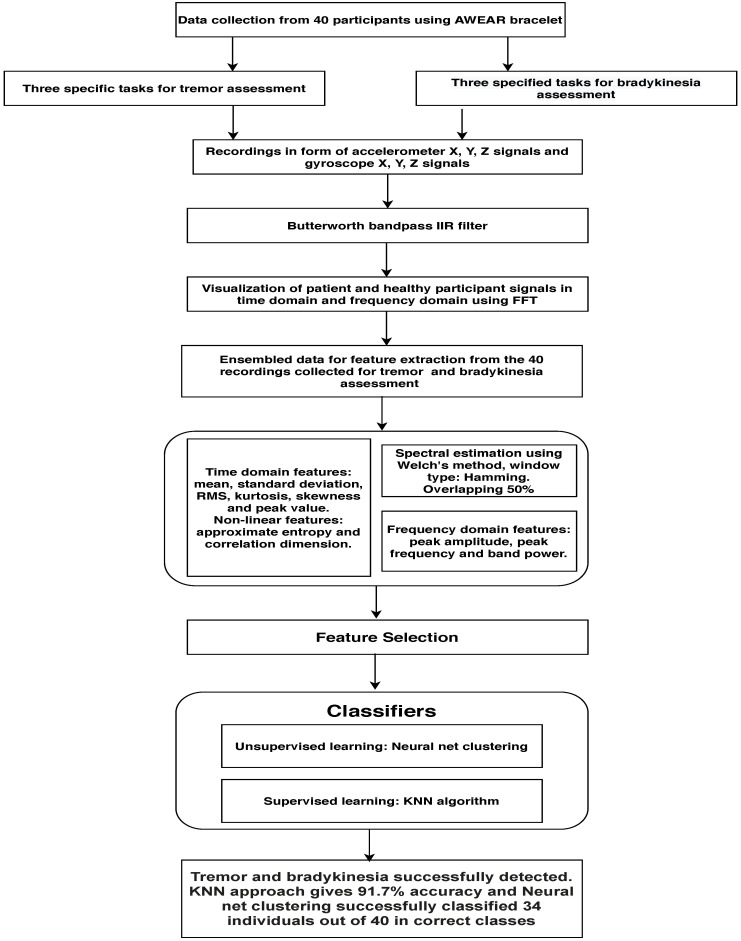
Illustration of the whole process for detection of tremor and bradykinesia.

As a result of the acquisition procedure, 40 recordings were collected—20 from healthy subjects, 10 recordings from patients who experienced tremor and 10 from patients having bradykinesia symptom. Initially, the data collected are processed by a filtering process to remove the drift, outliers and unwanted frequency, based on the study of previous research regarding what band the tremor frequency lies. Afterwards, the visualization is performed on data to enlighten the difference between healthy participants and the subjects who experience tremor and bradykinesia in their daily life. Afterwards, the features are extracted and machine learning (ML) classifiers performed classification for detection of tremor and bradykinesia. A generic flow diagram of the whole process is depicted in Figure 4.

### 3.1. Data Analysis

#### 3.1.1. Signal Processing

The sampling rate of the recorded signal is 100 Hz. The tremor frequency of upper extremities is lower than 13 Hz. Hence, as mentioned in [25], a 100 Hz sampling frequency is sufficient for PD-related motor features. Prior to visualization and feature extraction, all the recorded signals are filtered by a butterworth band pass IIR 10th order filter with cut-off frequencies of 2 Hz and 20 Hz.

#### 3.1.2. Signal Visualization

In order to analyze the signal, visualization is necessary to identify the main differences between signals of healthy participants and PD patients. It helps in removing the unwanted noise and frequencies, designing the accurate filter and also helps in understanding of signal performance in both the time and frequency domain.

### 3.2. Feature Extraction and Importance

To obtain accurate results, it is important to have proper features that define the characteristics of tremor and bradykinesia. As defined in Figure 4, we processed the collected data and ensembled it properly, from which we extracted both temporal and spectral features.

#### 3.2.1. Time Domain Features

Time domain features are divided into two parts, i.e., linear and non-linear features. The linear features which we extracted are as follows: mean, standard deviation, root mean square (RMS), kurtosis, skewness and peak value, while the non-linear features are the approximate entropy and the correlation dimension. We first computed the very basic statistics of signal, i.e., mean and standard deviation to check the regularity of signals. Afterwards, in order to get more insight, we extracted an impulsive metric, i.e., peak value. An impulsive metric helps in the analysis of the signal, while kurtosis and skewness are higher order statistics that aid the analysis of the behaviour of signals. Finally non-linear features were calculated in which the approximate entropy predicts the amount of unpredictability and the correlation dimension estimates the dimensions of samples. The combination of all theses features helps in determining the tremor and bradykinesia rhythm.

#### 3.2.2. Frequency Domain Features

In the frequency domain, first of all, the spectral estimation on time series signals is performed using Welch’s method by applying a hamming window in order to obtain a signal spectrum reflecting three dimensional (3D) tremor and bradykinesia movements before extracting spectral domain features. Welch’s method is also known as the periodogram approach, which is used for estimating power spectra by dividing the time series data into blocks by processing the periodogram in each block. In the frequency domain, another three features are calculated, which are the following: peak amplitude, peak frequency and band power. To extract these features, we defined the variable frequency bands with respect to the frequency at which tremor and bradykinesia movements occur.

### 3.3. Classification and Performance

An automatic classification for the detection of tremor and bradykinesia with respect to same-age elderly healthy adults based on the kinematic features (as explained in Section 3.2) is developed using unsupervised and supervised machine learning (ML) algorithms. All offline analyses were carried out using MATLAB R2016b (MATLAB, Mathworks, Natick, MA, USA).

#### 3.3.1. Using Unsupervised Method: The Neural Net Clustering Approach

**Figure 5:**
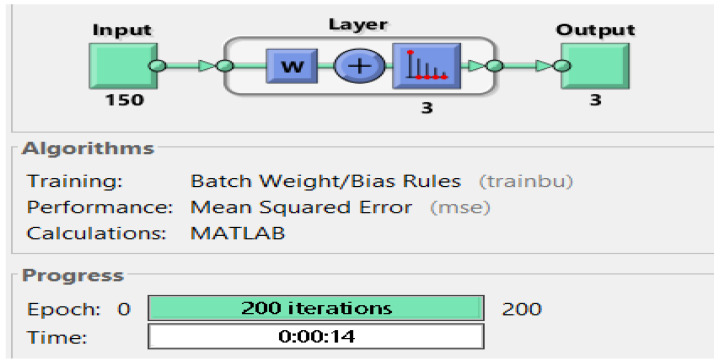
Neural network architecture.

Neural net clustering groups the data with similar characteristics and creates distinct clusters of it. This method uses self-organizing maps (SOM). A SOM is built with competitive layers which classify the dataset arranged in the form of feature vectors extracted from the samples collected from the participants. The architecture of the trained model is shown in Figure 5. The network is trained with the SOM batch algorithm. The map has been trained so that every neuron is used for one particular class. So, we classified three classes for which three neurons were chosen and the performance was computed based on the mean squared error (MSE).

#### 3.3.2. Using Supervised Method: The K-Nearest Neighbors (KNN) Approach

We used the KNN classifier for automatic assessment of tremor and bradykinesia in PD patients. It is a non-parametric approach which uses data and classifies the newest data points on the basis of proximity and similarity in the feature space.

## 4. Results

**Figure 6:**
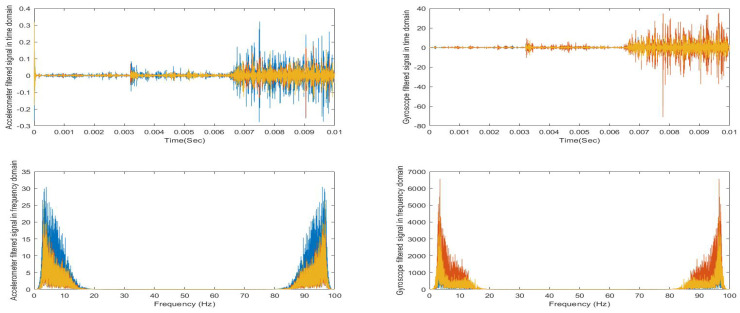
Visualization of healthy control signals before and after filtering in time and frequency domain.

**Figure 7:**
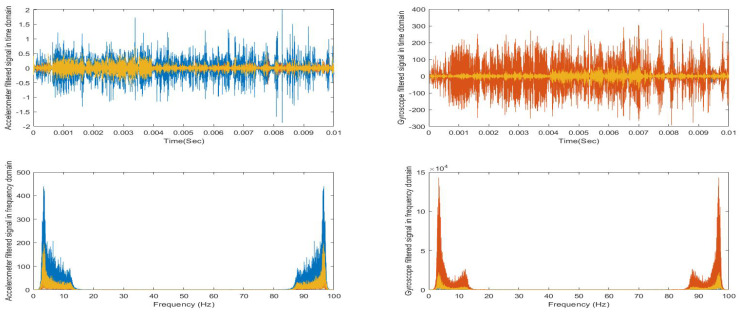
Visualization of PD patient signals before and after filtering in time and frequency domain.

As the study is based on sensors time series data, first we visualized healthy participant’s accelerometer and gyroscope raw signals in the time domain and then in the frequency domain by using fast fourier transform (FFT), as shown in Figure 6. This helped us to design a proper bandpass filter. Then, the filtered signals are visualized in time and frequency domain, as shown in Figure 7.

**Figure 8:**
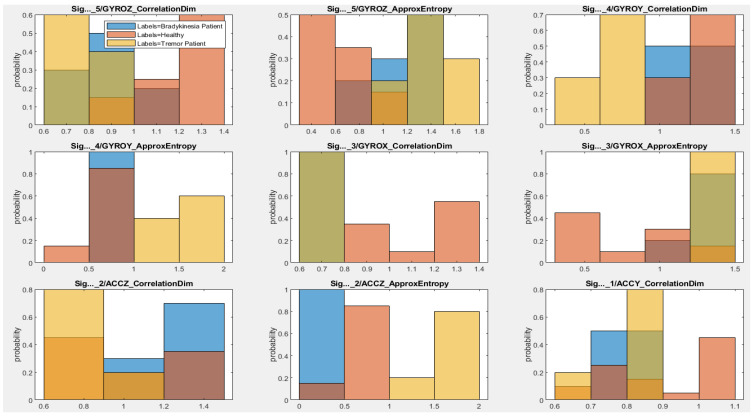

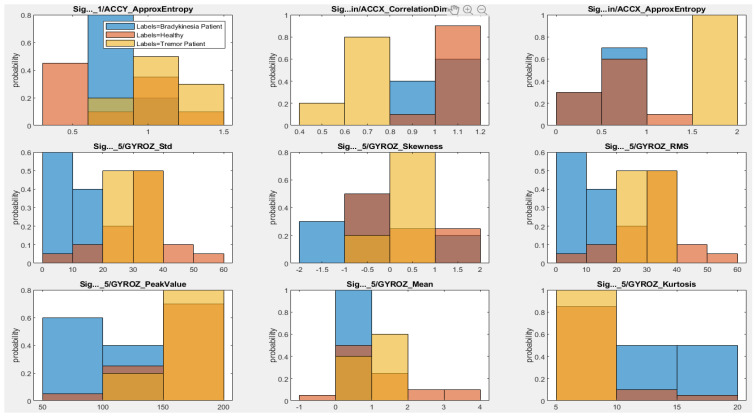
The histograms of the extracted features.

The effectiveness of the extracted features is evaluated using histograms, as shown in Figure 8. These histograms of each feature type show the correct distribution of features with respect to class type. On these plots, a different color indicates a different class type. Because of overlapping distribution of different class types and the high dimensionality of features, it is difficult to decide which features are more relevant and separable.

**Figure 9:**
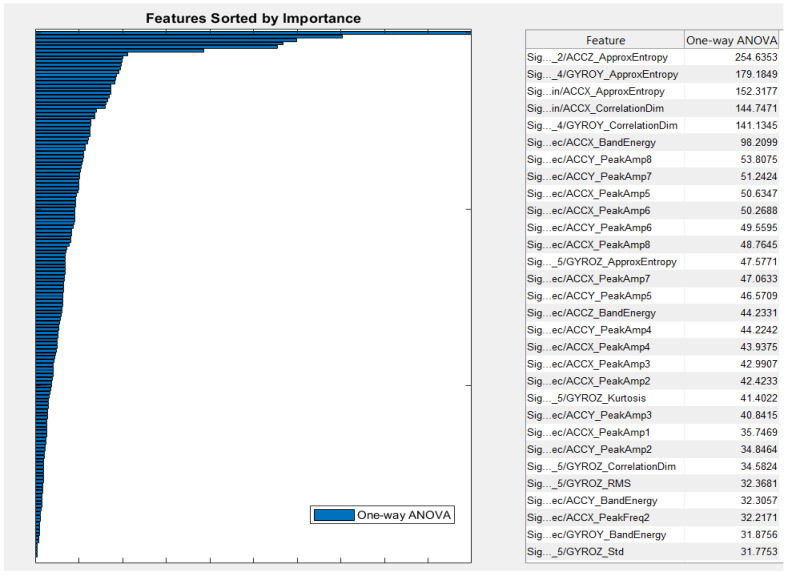
Features sorted by importance for the classifiers. The right side displays the ANOVA results, whereas the bars from the left side depict the normalized scores of different features.

The key finding of this research study is to figure out which parameters work well in distinguishing tremor and bradykinesia incidence from normal movements. For that, we performed a one-way ANOVA test to identify useful and distinctive features. Figure 9 shows the result of the one-way ANOVA approach. According to the results, the approximate entropy, correlation dimension, peak amplitude and band energies resulted from each axis of the accelerometer and the gyroscope had the highest values. Therefore, we selected the features which seemed more helpful in correctly diagnosising the tremor and bradykinesia incidents. The selected features with higher rank and importance are good candidates for training the NN clustering and the KNN models.

**Figure 10:**
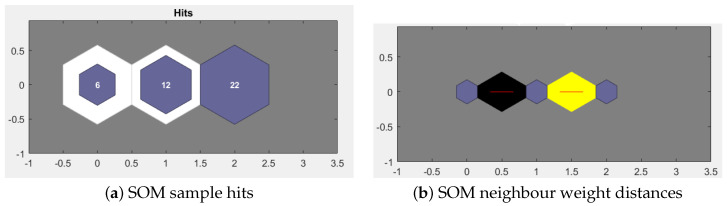
Results of the trained model.

The results of the neural net clustering trained model are shown in Figure 10a,b. Figure 10a emphasizes the sample hits, which means how many samples fall in each cluster. The cluster with the maximum samples is more covered with darker shade and less with whiter. Figure 10b shows SOM neighbour distances. The blue hexagons are the neurons and the red lines inside these neurons indicate the connection between neighbouring neurons. The colors in the areas containing the red lines shows the distances between neurons. The darker shades indicate larger distances and the lighter ones indicate smaller distances.

**Figure 11:**
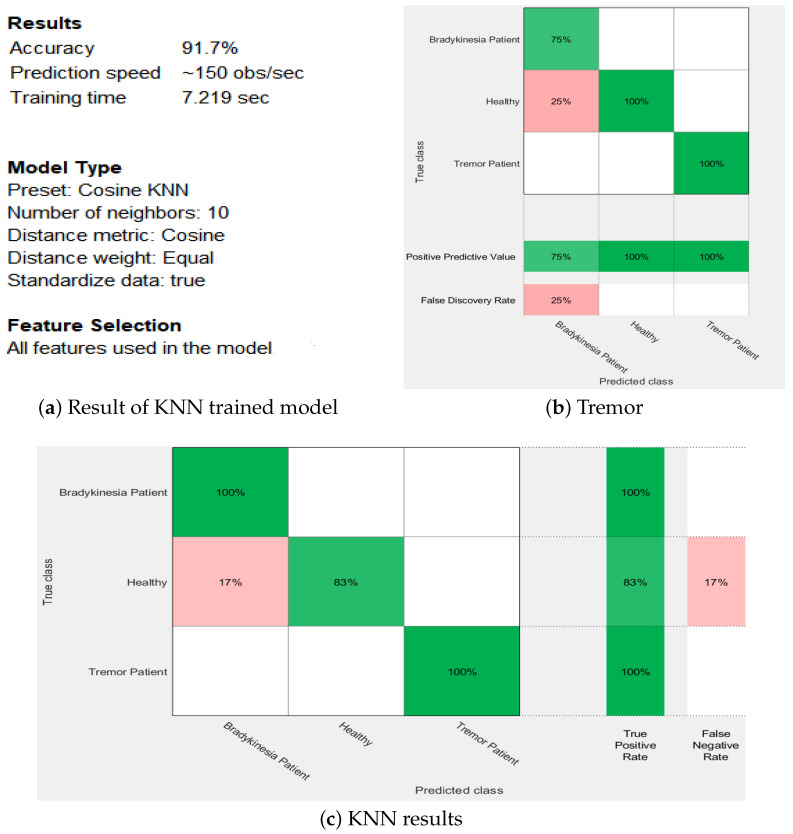
KNN classifier results.

Figure 11 shows the results of the KNN trained model with 91.7% accuracy. The number of neighbors is chosen as 10 with a hold-out validation of 30%. Figure 11a presents the accuracy and the number of neighbors used. Figure 11b,c show the confusion matrices which depict the performance of the trained model. In this context, Figure 11b shows the positive predictive values (PPV) and false discovery rate (FDR). The green-colored row correctly shows observations per the predicted class and the pink color indicates when the model has incorrectly predicted points in bradykinesia patients. Figure 11c offers the results for the true positive rates (TPR) and false negative rates (FNR). In the extreme last two columns, the green color shows how well the model performed and the pink color depicts the poor performance.

**Figure 12:**
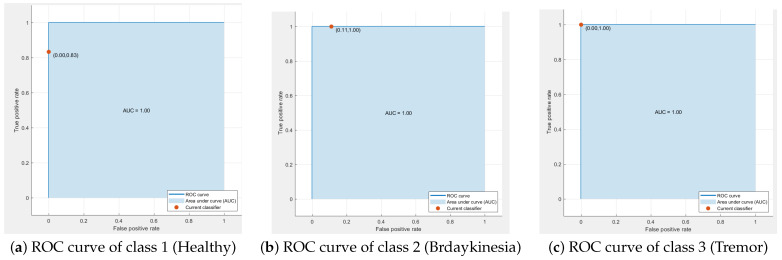
ROC curves.

Figure 12 elaborates the receiver operating curves (ROC) for each class, i.e., healthy, tremor and bradykinesia subjects. ROC curves are helpful in finding out sensitivity and specificity by using TRP and FPR. Figure 12a shows the ROC of the healthy participants, where TPR is 0.83 and FNR is 0.0. Figure 12b shows the ROC of the bradykinesia class, where TPR is 1.0 and FPR is 0.11 and Figure 12c presents ROC of the tremor class, where TPR is 1.0 and FPR 0.0. Table 5 describes the sensitivity and specificity of each class.

## 5. Discussion

This research study scrutinized the appropriateness of adopting an A-WEAR bracelet, which is developed using an accelerometer and gyroscope as an assessment tool for monitoring and diagnosis of PD tremor and bradykinesia. Particularly, three primary exercises followed by UPDRS criteria and clinically approved by practitioners were chosen for tremor detection, which involves the upper limb movements of the most effected hand and thus helps validate the bracelet for clinical use. In addition to the aforementioned exercises for tremor, three other main exercises for bradykinesia assessment, i.e., finger tapping, fist open and close and pronation/supination showed significant clinical association with neurologists’ assessments between two groups (healthy and patients subject).

To the best of our knowledge, no previous study has been done, as illustrated in Table 2 and Table 3, which uses the performance of multiple exercises for assessment of patients with PD. To test the potential of bracelet, we tried to extract the primary features and characteristics that distinguish two groups of participants. These features are further fused and entered into ML classifiers. Most of the classification problems typically involve very high dimensional features [45], resulting in complex classifiers and difficulties in training. Hence, our approach is to reduce dimensionality that eventually removes irrelevant, noisy and redundant features. We employed a one-way ANOVA approach that helped in feature reduction and maximized the relevance of the selected features to correctly distinguish tremor and bradykinesia as two different symtoms, unlike in [46], which used a one-way ANOVA approach to detect dyskinesia, tremor and bradykinesia as a single symptom. As per Figure 10, the most relevant features seem to be the approximate entropy, correlation dimension, peak amplitude and band energies, which act as input to the NN clustering and KNN classifiers.

From the last decade, it has been widely observed that in most of the published works, researchers showed great interest in involving ML classifier training over wearable inertial sensors data collected from PwPD [47] and tried to enhance the effectiveness and accuracy leaving behind the focus on the ability of the system to diagnose PD symptoms at an early stage. From the systematic review of [48], a high number of research works investigated solutions for lower limbs of PwPD and the it was concluded that there is a need to investigate motor assessment from upper limbs. For this reason, a solution in the form of an A-WEAR bracelet is proposed that provides the relevant and most significant features, which are classified using KNN and NN clustering methods, resulting in 91.7% accuracy and correct diagnosis of 34 out of 40 subjects. However, an A-WEAR bracelet does not show the correlation of features with UPDRS criteria to find out the exact correlation of mild to severe symptoms. In order to maximize the generalized quantification ability of this device, there is a need to make this device more portable, effective and flexible, keeping in mind that each gram of additional sensors added causes the peak frequency of tremor to decrease by up to 0.85 Hz, as discussed in [49], which ultimately decreases the acceleration amplitude. In addition to this, new data must be cross-examined and for that, more effective computational ML algorithms need to be analyzed. Conversely, no differences emerged regarding the gender of participants. Moreover as expected, the two groups differ in terms of globally approved motor disabilities, i.e, cognitive functioning.

## 6. Conclusions and Future Directions

The results of this research study prove that the AWEAR bracelet can be used for acquiring the right data that can determine a robust tremor and bradykinesia diagnosis. The bracelet does not weight much and is comfortable to wear for participants. The subjects performed some physical tasks with the hand, which is most affected by the disease. The data collected by the AWEAR device were visualized and analyzed and different temporal and spectral parameters were derived from them. To reduce the complexity of the classifiers, the key task was to find the importance of the parameters using the one-way ANOVA method. The results of one-way ANOVA represent the non-linear time domain features and the spectral features are more dominant than the rest of the linear features. Furthermore, utilizing the extracted parameters in ML classifiers, both in the KNN algorithm and neural network clustering, we were able to diagnose the PD patients’ cardinal symptoms from the healthy older adults with an impressive accuracy.

The main benefit of the extracted features and approaches employed in this research study is the interpretation of the results connected to a low-cost and easy to use wearable bracelet, which can be used without any assistance and in places where medical and financial resources are scarce. Likewise, the simplest tests can be conducted by participants and evaluated by a person without any experience.

In the near-future, the AWEAR bracelet will involve a cloud-based approach. It will also be improved with more advantageous features for remote diagnosis and determination of the disease progress. Considering clinical aspects, there is a continuous need for support from neurologists and PD patients, gathering more data and improving the ML models. Since this bracelet helps in PD identification, another goal is to refine the classes and to use it for identifying different tremor types, tremor severity levels and bradykinesia.

## Figures and Tables

**Table 1:** The key features of Parkinsonian tremor, dystonia tremor and essential tremor.

Key Features	Parkinsonian Tremor	Dystonia Tremor	Essential Tremor
Frequency	4–6 Hz	7 Hz	4–8 Hz
Amplitude	Regular	Irregular	Regular
Symmetry	Asymmetrical	Asymmetrical	Symmetrical
Topography	Hands > other	Head > hands > others	Hands > head > voice > others
Potential accompanying sign	Bradykinesia, rigidity	Dystonic posture	Impaired tandem gait
Suppression of tremor during movement onset	In most cases	Rare	Not found
Activation condition	Rest > postural/kinetic	Postural > kinetic > rest	Postural > kinetic > rest
Sensory tricks	No	Yes	No
Handwriting	Micrographia	Macrographia	Large angulated loops
Decreased arm swing	Yes	May be in dystonic limb	No

**Table 2:** Studies on tremor.

Study [Ref]	Technology Description	Location	Subjects	Algorithms	Metrics	Activity	Main Results
[17]	IMU unit. Six-axis inertial sensor on index finger of tremor dominant hand	Hospital	35 PD patients and 22 ET	Autoregression process using Yule-walker method and *t*-tests.	Power spectrum of subsequences, peak frequency	3 tasks each of 10 s, i.e., kinetic, postural and resting tasks	Temporal fluctuation of resting task can differentiate between PD and ET
[22]	4 inertial sensors taped on hands, feet and around the waist	Clinical	7 PD patients	Wilcoxon’s two-tailed rank sum test, bonferroni correction and spearman’s rank correlation coefficient testing	Angular velocity and power spectral density	Two tests. Rest tremor, while sitting at rest patient was reading a text aloud for 45 s. For action tremor a tapping movement performed for 30 s	Application of DBS come forth in a redistribution of power in the tremor and LF band
[23]	Sensors at 6 different positions of subject’s body i.e., right and left wrists (RW and LW), right and left legs (RL and LL), waist and chest	Clinical	18 PD patients and 5 HS	Hidden Markov’s model	Angle between two sensors and LF energy. For tremor severity classification: spectrum entropy, LF and HF energy, ratio of high to total energy and energy from other body segments	DLA’s	(1) Quantifies tremor severity with 87% accuracy (2) Discriminates tremor from other PD symptoms.
[26]	IMU	Hospital	7 PD patients	Least square estimation models	Amplitude of parkinsonian tremor and dominant frequency of parkinsonian tremor	3 tasks. Rest tremor (RT), postural tremor (PT) and action kinetic tremor assessment (KT). Each last for 10 s.	Measured amplitude correlated well with judgement of neurologists (r = 0.98)
[24]	Kinesia affixed finger worn sensors and wrist worn command module	Clinical	60 PD patients	Multiple linear regression model	Peak power frequency of peak power, RMS of angular velocity and RMS of angle	RT assessed for 30 s when participant remain settle with his hands still in lap, PT for 20 s with arms stretched out infront and KT while participant frequently enlarged his arm and touched his nose for 15 s	Quantitative kinematic features are processed and highly correlated to clinicians scores
[28]	Part 1: 3 uni-axial accelerometers on one wrist. In part 2: same as of part 1 also 2 pairs of uni-axial accelerometers (at stemum and upper dominant leg)	Part 1 in lab and part 2 in home	Part 1: 7 patients, part 2: 59 patients and 43 HS	Part 1: FTFT, detect tremor if longer than minimal duration (1.5 s) of dominant frequency with limited BW. Part 2: same as P1 also determine standing vs. sitting based on gravitational vector	Part 1 measured amplitude, dominant frequency duration and BW. Part 2: same as P1 also measured duration of posture of tremor and mean amplitude	In part 1 seated postures recorded at rest and while performing motor activities. In part 2 measured for 24 h while keeping diary	Part 1: Tremor vs. no tremor compared to specialists: SENS > 82%; SPEC > 93%. Part 2: Duration of tremor moderately correlated with UPDRS score for resting tremor (ϱ = 0.66 standing, 0.77 sitting) Intensity of tremor correlated with resting tremor (ϱ = 0.70 standing, 0.75 sitting)
[30]	Part 1: 3 uni-axial gyroscopes near wrist and part 2: two uni-axial gyroscopes near wrist	Hospital	7 PD patients	IIR filter with 3 s windows and autoregression model. Tremor detected if frequency lies between 3.5 and 7.5 Hz and amplitude >0.92. Tremor amplitude estimated from RMS angular velocity	Dominant pole frequency and amplitude	45 min of 17 ADL while videotaped (DBS on and DBS off). In second part 3–5 h moving freely	Tremor vs. no tremor compared: SENS = 99.5%, SPEC = 94.2%. Estimated tremor amplitude from roll axis showed high correlation (r = 0.87) to the UPDRS tremor subscore.
[19]	For EMG, electrodes at belly and ME6000-biosignal monitoring system is used. Tri-axial accelerometers attached to palmar sides of subjects wrists	Hospital	42 patients and 59 HS	K-means algorithm	Kurtosis variable of EMG (K), crossing rate variable of EMG (CR), correlation dimension and recurrence rate of EMG, sample entropy of acceleration (SampEn), coherence variable of EMG and acceleration (Coh)	Subjects asked to hold their elbows at 90° angle for 10–30 s	According to clustering results one cluster contained 90% HC and two other clusters 76% of patients
[21]	Data from gyroscope and accelerometer	Clinical	23 PD patients	To analyze correlation pearson correlation is used	Acceleration vector and rotation rate vector	Wearing iphone on top of hand while sitting on chair and resting both hands on lap atleast for 30 s. Repeated for both hands	Strong correlation (x > 0.7 and *p* < 0.01) between patients UPDRS score and signal metrics applied to measure signal

**Table 3:** Studies on Bradykinesia.

Study [Ref]	Technology Description	Location	Subjects	Algorithms	Metrics	Activity	Main Results
[38]	Pairs of uni-axial accelerometers on sternum, upper leg, and wrist	Hospital	NA	Discriminant analysis to determine thresholds, Multiple regression analysis for objective measures and UPDRS scores	Bradykinesia: magnitude of acceleration for arm and leg; Hypokinesia: MIP (period with acceleration below a threshold) for hand and trunk	24-h continuous recording	Bradykinesia: mean arm and leg accelerations showed inverse relation with UPDRS (R2 = 0.1, R2 = 0.45)
[39]	Tri-axial accelerometers near the wrists, ankles and hip	Main room for a day program of PD	2 PD patients	Classification trees and neural networks	Absolute value of derivative of magnitude of acceleration, position and magnitude correlation between sensors	2 subjects recorded for about 320 min each while videotaped	Bradykinesia/ hypokinesia vs. no bradykinesia/ hypokinesia compared to neurologist: Neural network with c-index of 88.0–92.1% Classification tree with accuracies of 74.8–85.3%
[40]	Tri-axial accelerometers on upper arms, forearms, supper thighs, and shins	Lab	12 PD patients	Clustering evaluation index to select features and linear discriminant classifier to predict performance of features	Intensity (RMS), auto-covariance, dominant frequency, correlation features, and entropy	Standardized clinical motor tasks (alternating hand movements, finger to nose, and heel tapping) while videotaped	Best features: approximate entropy and intensity (RMS of acceleration) Optimal window length 6 s
[15]	9 DoF sensor (3 accelerometers, 3 gyroscopes and 3 magnetic sensors). On the dorsal side of the index finger, dorsal side of the forearm close to the wrist and on the in step of the foot over the shoe of the participant.	Clinical	25 PD patients and 10 HS	SVM	Mean, amplitude and mean frequency	finger tapping, diadochokinesis and toe tapping	The classification errors for finger tapping, diadochokinesis and toe tapping were 15–16.5%, 9.3–9.8% and 18.2–20.2% smaller than the average inter-rater scoring error

**Table 4:** Demographic and clinical details of healthy control and patient with Parkinson disease.

Healthy Control	Patient with PD			
Age (Gender)	Age (Gender)	UPDRS (0–56)	H & Y (1–5)	Disease Duration (Years)
75(F)	62(F)	23	1.5	7
64(M)	66(F)	5	1.5	6
75(M)	72(F)	9	2	6
80(F)	73(F)	26	2	20
83(M)	78(M)	5	1	13
65(M)	65(M)	27	1	14
65(M)	79(F)	23	1	5
61(M)	69(F)	15	2	3
63(M)	80(M)	25	2	8
70(M)	81(M)	18	1.5	4
70(F)	60(F)	20	2	11
76(M)	80(F)	26	2	10
67(M)	65(F)	7	1	1
66(F)	75(M)	30	1	2
62(M)	72(F)	18	1	7
66(M)	63(F)	22	1.5	3
74(M)	66(F)	15	1.5	9
71(M)	83(F)	15	2.5	10
72(M)	75(F)	32	2.5	5
80(M)	69(F)	30	2.5	10
70.25(±6.307)	71.65(±6.872)	18.91(±7.831)	1.65(±0.526)	7.7(±4.495)

**Table 5:** Sensitivity and specificity of each class.

Class	Sensitivity	Specificity
1 (Healthy)	0.83	1.00
2 (Bradykinesia)	1.00	0.89
3 (Tremor)	1.00	1.00

## Data Availability

Not applicable.

## Abbreviations

The following abbreviations are used in this manuscript:

UPDRS	Unified Parkinson’s Disease Rating Scale
PD	Parkinson Disease
QoL	Quality of Life
ADL	Activities of Daily Life
LF	Low frequency
HF	High frequency
SVM	Support vector machine
HMM	Hidden markov model
DBS	Deep brain simulation
ET	Essential tremor
KNN	K nearest neighbours
PT	Postural tremor
KT	Kinetic tremor
SOM	Sample of map
PwPD	Patients with Parkinson Disease
